# Academic medicine career intentions among medical residents: a social cognitive career theory approach

**DOI:** 10.1186/s12909-026-09740-4

**Published:** 2026-06-19

**Authors:** Aurore Deledalle, Valentine Bour, Jean-Michel Galharret, Élisa Sarda, Pierre-Antoine Gourraud, Fleur Cohen, Patricia Lemarchand, Anne Congard

**Affiliations:** 1 Nantes Université, Univ Angers, Laboratoire de Psychologie des Pays de la Loire, LPPL UR 4638, Nantes, F-44000 France; 2 StatSC, INRAE, ONIRIS, Nantes, France; 3 L-VIS Laboratory of Vulnerabilities and Innovation in Sport (UR 7428), Université Claude Bernard Lyon 1, Lyon, France; 4 Nantes Université, CHU de Nantes, INSERM, CIC 1413, Pôle Hospitalo-Universitaire 11 : Santé Publique, Clinique des données, Nantes, F-44000 France; 5 Service de Médecine Interne 2, Centre des Maladies Infectieuses et Immunologique INSERM UMRS 1135, Sorbonne Université, Assistance Publique Hôpitaux de Paris, Hôpital de la Pitié-Salpêtrière, Paris, France; 6 Nantes Université, CHU Nantes, CNRS, INSERM, l’Institut du thorax, Nantes, F-44 000 France

**Keywords:** Academic medicine, Social cognitive career theory, Career development, Research interest, Gender differences, Self-efficacy

## Abstract

**Background:**

Academic medicine (AM) careers combine research, teaching, and patient care, yet their attractiveness has declined in recent years, particularly among women, who remain underrepresented in this field. Previous research highlights the central role of interest in research, but the cognitive and contextual processes underlying academic career intentions during residency remain insufficiently understood. Social Cognitive Career Theory (SCCT) provides a relevant framework for examining how individual cognitions and learning experiences jointly shape career intentions.

**Methods:**

This cross-sectional study included 1,570 French medical residents who completed an online questionnaire. Guided by the SCCT framework, we examined the relationships between learning experiences (academic satisfaction and perceived stress), self-efficacy, outcome expectations (operationalized through work centrality), professional interest in research, and intention to pursue a career in academic medicine. Hierarchical ordinal regression models were used to predict AM career intention, and structural equation modeling was conducted to investigate the formation of professional interest in research. Gender and prior research experiences (master’s or PhD) were examined as moderating factors.

**Results:**

Overall, residents reported low intention to pursue a career in academic medicine, with women expressing significantly lower intentions than men. Professional interest in research emerged as the strongest predictor of AM career intention, followed by research experiences and mentorship. Outcome expectations, measured through work centrality, also contributed significantly to career intentions. Structural equation modeling supported key assumptions of SCCT: perceived stress was negatively associated with self-efficacy, academic satisfaction was positively associated with outcome expectations, and work centrality played a central role in shaping professional interest in research. Gender differences in career intentions were largely explained by disparities in learning experiences and research opportunities rather than by differences in outcome expectations. Moderation analyses suggested gender-specific patterns at advanced levels of research involvement.

**Conclusions:**

These findings support the relevance of SCCT for understanding academic medicine career intentions and highlight the central role of professional interest in research and contextual learning experiences. Promoting positive learning environments, providing early and structured research exposure, and ensuring access to mentorship may represent key levers for fostering academic career engagement and addressing gender disparities in academic medicine.

**Supplementary Information:**

The online version contains supplementary material available at https://doi.org/10.1186/s12909-026-09740-4.

## Introduction

A career in academic medicine (AM) involves a combination of research, teaching, and patient care, and encompasses both attractive and less appealing aspects. These aspects relate to the nature of academic missions, individuals’ lived experiences (e.g., work–life balance, career satisfaction), and employment-related conditions such as the work environment [1]. Despite the diversity and potential appeal of these careers, their overall attractiveness has declined in recent years [2]. At the same time, although women now represent a growing proportion of medical students and physicians, they remain markedly underrepresented in academic medicine [3].

One of the most important characteristics of a career in AM is a strong commitment to research. Previous studies suggest that an interest in research plays a decisive role in whether residents pursue an academic career. Two literature reviews [4, 5] indicate that completing a medical degree alongside a graduate degree (master’s or PhD), enrolling in research-oriented or fellowship programs, and participating in research activities are strongly associated with later involvement in academic medicine. Recent initiatives have also highlighted the importance of structured training programs in supporting the development of physician-scientists [6]. Understanding how interest in research develops during medical training therefore appears essential for explaining career intentions in AM. However, existing studies have mainly focused on isolated predictors, without fully examining how these factors interact in shaping career intentions.

A substantial body of literature has examined factors influencing interest in academic careers and has highlighted gender differences in this process. Some studies report that women express lower interest in research compared with men [7]. This difference has often been interpreted through gendered orientations toward occupations, whereby men are thought to favor more object-oriented activities, while women are more drawn to people-oriented professions such as care or counseling [8]. Beyond individual preferences, gender differences in academic career interest are increasingly understood as the result of interacting individual, social, and structural factors, including gender norms, organizational culture, and unequal access to opportunities in academic environments [7, 9–12]. In particular, previous studies have highlighted the role of mentoring, institutional climate, and persistent gender inequalities in shaping career trajectories in academic medicine [9, 11]. Taken together, these findings highlight that both individual preferences and structural factors contribute to gender differences in academic career interest, but are often examined separately. This fragmentation points to the need for an integrative framework capable of capturing the interaction between cognitive and contextual factors in the development of career intentions.

In career guidance psychology, several theoretical models have been developed to explain how career interests and intentions are formed [13–15]. Among them, Social Cognitive Career Theory – SCCT [16] is one of the most widely used. SCCT assumes that career development is shaped by the interaction of three core cognitive variables: self-efficacy beliefs (individuals’ beliefs in their capacity to perform specific activities), outcome expectations (beliefs about the consequences of engaging in those activities), and goals (such as the intention to pursue a particular career). In addition, the model emphasizes the role of contextual supports and barriers that may facilitate or constrain career-related processes.

SCCT provides a particularly relevant framework for examining interest in research and career intentions in academic medicine, as it allows for the integration of both individual cognitive factors and contextual experiences, such as mentoring opportunities, perceived stress or discrimination during medical education. This model has been successfully applied in fields facing recruitment challenges similar to those observed in AM, such as science, technology, engineering, and mathematics (STEM) disciplines. For example, Inda et al. [17] investigated gender differences within the SCCT framework among engineering students and found that men reported higher self-efficacy and stronger interest in engineering-related activities than women, although outcome expectations did not differ significantly by gender.

Within the context of academic medicine, Bakken et al. [18] highlighted the conceptual relevance of SCCT for understanding academic career intentions, without empirically testing the model. Subsequent studies have primarily used SCCT to assess the effects of specific research training programs on self-efficacy among men and women [19]. Only one study has applied SCCT to career development in academic medicine more broadly, focusing on students enrolled in degree programs, PhD tracks, or fellowship positions [20]. However, participation in structured research programs during residency is not universal, limiting the generalizability of these findings to all medical residents. This integrative perspective addresses the limitations of prior research that tended to examine these dimensions separately.

As a whole, these findings suggest that interest in research and academic career intentions cannot be fully understood without taking into account both individual cognitive processes and contextual learning experiences, as proposed by the SCCT. The present study aims to apply the SCCT framework to better understand the processes through which medical residents develop an interest in research during their training. Our model integrates factors consistently identified in the literature, such as research experiences and mentoring, while accounting for their interaction with individual cognitive variables, including perceived stress during studies, academic satisfaction, work centrality, and general self-efficacy. Gender is examined as a central dimension shaping this process.

Building on these gaps, the present study aims to provide a more integrative understanding of academic career intentions among medical residents. Specifically, this study pursues three objectives. First, we examine whether the core variables of SCCT contribute to explaining residents’ intention to pursue a career in academic medicine (objective 1). Second, we investigate the relationships among the variables involved in the development of professional interest in research (objective 2). Third, we explore the role of gender and the influence of prior research experiences on these cognitive and motivational processes (objective 3).

**Fig. 1:**
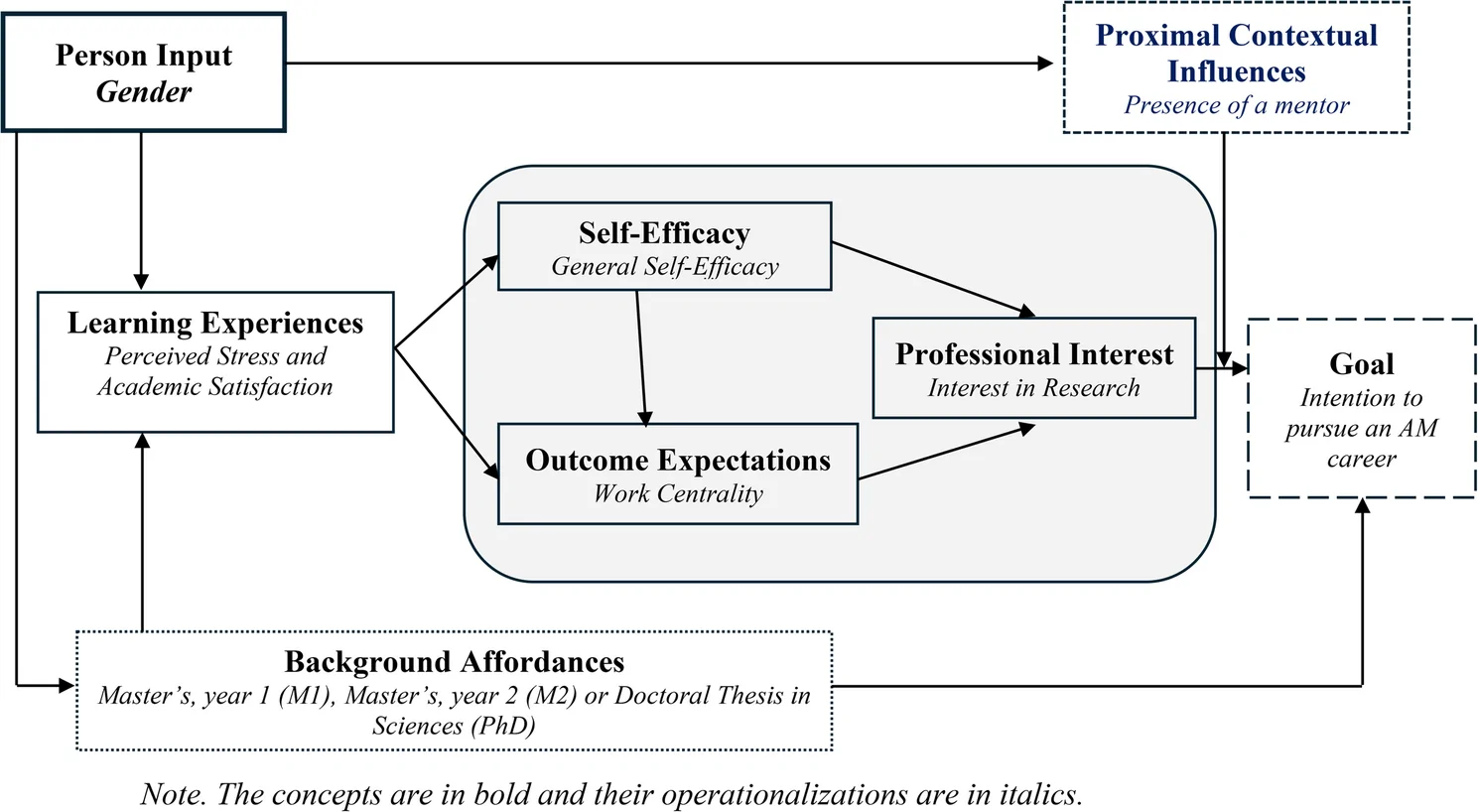
Model of personal, contextual, and cognitive factors affecting behavior related to career choice

The hypothesized relationships between the different variables are presented in Fig. 1: the primary factor influencing the intention to pursue an AM career is likely to be professional interest in research. The literature review indicates that exposure to research through the completion of a master’s or a PhD (background affordances) and the presence of a mentor (contextual influence) also have an impact. Furthermore, an individual’s cognitive variables from Lent’s model (self-efficacy, outcome expectations) and learning experiences (academic satisfaction and perceived stress) are expected to enhance the predictive model for AM career intention.

Beyond these objectives, this study makes several contributions to the literature. First, it represents one of the first empirical applications of the Social Cognitive Career Theory (SCCT) framework to academic medicine career intentions among medical residents, and, to our knowledge, the first within the French context. Second, by integrating cognitive variables and contextual learning experiences within a single model, it provides a more comprehensive understanding of how professional interest in research is formed during residency. Third, this study highlights the moderating role of gender and research experience, revealing differential patterns in the development of research interest at more advanced stages of academic involvement. Together, these contributions offer a novel and contextually grounded perspective on academic career development in medicine.

## Method

### Sample

A total of 1,570 participants were included in the final analyses. Among initial respondents, 104 were excluded due to insufficient responses on the professional interest scale, and 3 non-binary participants were excluded due to the focus on gender comparisons. The main characteristics of the sample are summarized in Table 1. Participants were predominantly women (63.5%) with a mean age of 27.28 years (SD = 2.61). The majority did not have children (93.6%). Residents from all years of training and a wide range of specialties were represented, with most participants enrolled in medical specialties. Regarding research background, most residents had limited prior research experience, and less than half reported having a mentor. Participants were recruited from across France, including a substantial proportion from the Île-de-France region^1^.

**Table 1:** Characteristics of the study sample (*N* = 1,570)

Variable	Category	*n* (%)
Gender	Women	997 (63.5%)
	Men	573 (36.5%)
Age (years)	Mean (SD)	27.28 (2.61)
Children	Yes	100 (6.4%)
	No	1,470 (93.6%)
Year of residency	Year 1	314 (20%)
	Year 2	330 (21%)
	Year 3	361 (23%)
	Year 4	361 (23%)
	Year 5	204 (13%)
Specialty	Medical specialties	1,108 (70.6%)
	General practice	223 (14.2%)
	Surgical specialties	104 (6.6%)
	Other	135 (8.7%)
Research experience	None / M1	1,243 (79.2%)
	M2 / PhD	327 (20.8%)
Mentor	Yes	639 (40.7%)
	No	931 (59.3%)
Region	Île-de-France	418 (26.6%)
	Other regions	1,152 (73.4%)

The majority of residents either have no background experiences of research or have only completed their first year of a master’s (M1) (79.2%). A higher proportion of women have no background experiences compared to men (38.3% versus 29.0%, χ² = 13.57, *p* < .001). In the second year of master’s (M2), the proportion of men is significantly higher than that of women (26.4% versus 17.6%, χ² = 16.60, *p* < .001), whereas in M1, these proportions were equal (around 44%, χ² = 0.02, *p* > .05). In addition, in comparison to women, a higher percentage of men indicate having a mentor (44.5% versus 38.5%, χ² = 5.16, *p* < .05).

### Procedure

Participants were recruited through social media using online advertisements or email blasts without any remuneration. As recruitment was conducted through open dissemination (social media and email distribution), the total number of individuals who received the survey invitation could not be determined, and therefore no response rate could be calculated. This recruitment strategy may have introduced self-selection bias. Participants completed the questionnaire anonymously via Lime Survey. All procedures were conducted in accordance with the ethical standards of the Ethics Committee for Non-Interventional Research (CERNI) of Nantes University (ethics committee approval no. 26072022). This study is part of a broader research initiative within the framework of the PULWAR research project. Pre-registration is available at https://osf.io/9yseq/?view_only=1c72743f542b402ba67beed6908e597d.

The participant selection process is summarized as follows: respondents completed the online questionnaire, and exclusions were applied based on predefined criteria (see above).

### Measures

The questionnaire was specifically developed by the authors for this study, based on the Social Cognitive Career Theory framework. The full version of the questionnaire is provided as Supplementary Material. Prior to data collection, the questionnaire was pilot-tested with two participants (a hospital-university assistant and a PhD candidate) using cognitive interviewing techniques. This phase aimed to assess item clarity, relevance, and interpretability, and led to minor revisions to improve wording, response options, and overall comprehensibility. 

#### Goal measure (SCCT): intention to pursue an AM career

In Lent’s model, the goal is defined as “*the type of activity domain one wishes to pursue*” [20, 21]. In this study, participants were asked to indicate their level of intention to pursue an AM career (1 = Not interested at all; 2 = Slightly; 3 = Moderately; 4 = Fairly; 5 = Very interested).

#### Professional interests (SCCT): interests in research

The *Attitude toward Research Scale *[22] was used to measure professional interest in research. We retained the “Interest” subscale, which focuses on intrinsic engagement with research activities and showed good internal consistency in the original study (α = 0.856) and in the present study (α = 0.819). No modification of the original items was performed; only the “Interest” dimension was selected based on its theoretical relevance to the SCCT framework and its alignment with the construct of professional interest in research. Although the scale was originally developed among teacher candidates, the selected subscale assesses a general interest in conducting research and integrating research activities into one’s professional identity, rather than attitudes specific to teaching. Because the items focus on engagement with research as a professional activity, we considered them conceptually applicable to medical residents and consistent with the SCCT construct of professional interest in research. The scale consists of eight items that assess professional interest in research in a broader sense, evaluating a liking for engaging in research activities and integrating it as an essential part of a career path (e.g. “*I like doing research*”, or “*Research is indispensable in my professional life*”). Participants rated their responses on a 5-point Likert scale ranging from “Strongly disagree” (1) to “Strongly agree” (5) with an optional response “Does not apply”. Respondents who answered fewer than 75% of the items on the scale, i.e., more than two items with the response “Does not apply”, were excluded from the study (*N* = 104), because their overall score on professional interest in research would not have been representative of this measure.

#### Outcome expectations (SCCT): work centrality

Within the SCCT framework, outcome expectations refer to individuals’ beliefs about the anticipated consequences of engaging in a given activity. In this study, outcome expectations were operationalized through work centrality, which refers to the extent to which the individual believes that involvement at work will bring them benefits. While outcome expectations are often operationalized in SCCT studies through anticipated extrinsic or intrinsic rewards, we chose work centrality as it captures the extent to which individuals expect work to provide meaning, identity, and personal value. This operationalization is particularly relevant in academic medicine, where strong professional commitment and engagement are central characteristics of the career. High work centrality indicates high outcome expectations toward work. The scale includes five items asking for the degree of agreement with statements about what people want for their future professional life (1 = Strongly disagree, to 5 = Strongly agree) (e.g. “*I feel that my sense of worth will come through my work and the strength of my commitment*” and “*I desire for my work to shape my identity and radiate into all areas of my life*”). 

#### Self-efficacy beliefs (SCCT): general self-efficacy

Within the SCCT framework, self-efficacy beliefs refer to individuals’ confidence in their ability to successfully perform specific tasks and cope with challenges in a given domain. In this study, self-efficacy beliefs were assessed using the General Self-Efficacy Scale (GSE; [23]), a 10-item self-reported instrument measuring optimistic self-beliefs (e.g., “I can always manage to solve difficult problems if I try hard enough”). Participants rated their agreement on a 4-point Likert scale ranging from “Not at all true” (1) to “Exactly true” (4). Internal consistency was satisfactory in the present study (Cronbach’s α = 0.827).

#### Learning experiences (SCCT): academic satisfaction and perceived stress

In SCCT, learning experiences constitute a central mechanism through which person inputs and background contextual affordances shape self-efficacy beliefs and outcome expectations. These experiences are not only defined by objective educational pathways but also by individuals’ subjective perceptions of their training context and demands.

Perceived stress was assessed using the Short Perceived Stress Scale (PSS), a 4-item short form derived from the original 14-item scale [24]. Participants responded using a 5-point Likert-type scale ranging from 1 (Almost never) to 5 (Always). Internal consistency was satisfactory (Cronbach’s α = 0.834).

Academic satisfaction was measured using the Scale of Satisfaction with Studies (SSS), a 5-item self-reported instrument assessing individuals’ satisfaction with their training environment and academic trajectory. Satisfaction refers to an “affinity for the larger settings in which activities are situated” [25]. Items were rated on a 7-point Likert-type scale ranging from 1 (Strongly disagree) to 7 (Strongly agree).

Together, these two measures capture both negative (perceived stress) and positive (academic satisfaction) aspects of residents’ learning experiences, in line with the SCCT conceptualization of experiential sources influencing career-related cognitions and interests.

#### Background affordance (background experiences of research) and person inputs (gender)

Within the SCCT framework, background contextual affordances and person inputs are conceptualized as interacting influences. Background Affordances are assessed by additional learning experiences during the residency through a degree (master’s year 1, master’s year 2, or PhD). These background experiences of research provide opportunities to gain mastery experiences. The person input is gender (“*You are: a man/a woman/non-binary*”).

Other socio-demographic questions were included to characterize our sample (specialty, age, year of residency, medical school location, number of children…).

#### Proximal environmental influences (SCCT): mentorship

Within the SCCT framework, proximal environmental influences refer to contextual factors that directly shape career-related choices at critical decision points. In the present study, mentorship was considered a key proximal contextual influence and was assessed using a dichotomous (yes/no) item: “Within your professional circle, do you have a mentor (a knowledgeable and experienced guide or advisor, someone you hold in high esteem, whom you can emulate, and/or who provides you with their expertise and experience)?”.

All scales showed satisfactory internal consistency (Cronbach’s α ≥ 0.80). To further assess the quality of the measurement model, standardized factor loadings, Composite Reliability (CR), and Average Variance Extracted (AVE) were calculated for all latent variables. The results are presented in Table 2 and support the reliability and convergent validity of the constructs.

**Table 2:** Psychometric properties of the latent constructs: standardized factor loadings, AVE, and McDonald’s omega

	Standardized factor loadings range	Average variance extracted (AVE)	Reliability (ω)
Professional Interest in research	[.39;.90}	.439	.860
Work Centrality	[.52 ;.79]	.484	.788
General self-efficacy	[.52 ;.85]	.533	.903
Perceived stress during studies	[.74 ; .86]	.625	.844
Academic Satisfaction	[.72 ;.84]	.632	.884

### Data analysis

Analyses were conducted in line with the objectives derived from the SCCT framework. First, we examined the predictors of intention to pursue a career in academic medicine using hierarchical ordinal regression models (objective 1). We investigated the association of the intention to pursue an AM career with perceived stress during studies, academic satisfaction, self-efficacy, work centrality and professional interest in research. Moreover, we studied whether this association differs according to gender and background experiences (objective 3). Since the intention to pursue an AM career is measured on an ordinal scale, a common method in such studies is to use this type of model. For these models, the deviance is used to test the significance of the predictors and/or the blocks of predictors. Several definitions of the proportion of variances (i.e. R^2^ in a linear model) have been proposed for these models and are known as pseudo-R^2^. In the following, we choose the likelihood ratio R2L introduced by Cohen, since it is the most analogous to R^2^ for linear regression, with variance reduction replaced by deviance reduction.

Second, to investigate the cognitive and motivational processes underlying professional interest in research (objective 2), we tested a structural equation model. These models make it possible to highlight the indirect effects of learning experiences on professional interest in research through self-efficacy and outcome expectations. Moreover, the moderating role of gender and background experiences in these associations was assessed using these models (objective 3). To assess measurement model qualities, the indices used include the Comparative Fit Index (CFI), the Tucker Lewis Index (TLI), the Root Mean Squared Error of Approximation (RMSEA) and the Standardized Root Mean Squared Residual (SRMR).

Statistical analyses were performed using R software (Version 4.3.0, 2023). The structural equations were modeled using the R package lavaan [26].

## Results

### Descriptive statistics

Descriptive statistics were first calculated for the variables in the SCCT model and their correlations (see Table 3), then the dependent variable of the model, the intention to pursue an academic career in medicine, was examined for the whole sample and according to gender. As expected, on the whole, residents are not very interested in an AM career: 38.2% fall into the “Not at all” category (1), 41.7% in “Somewhat the intention” (2–3) and 20.1% in “Strong intention” (4–5). Women are over-represented in the “Not at all” category (43.2% of female respondents versus 29.3% of male respondents, χ² = 29.25, *p* < .001) and under-represented in the “Strong intention” category (15.1% versus 28.8%, χ² = 41.33, *p* < .001).

**Table 3:** Correlation and descriptive statistics (sensitivity and reliability) of Lent’s core variables

**Variables**	**1**	**2**	**3**	**4**	**Mean** (***SD***)	**McDonald’s**
Perceived Stress during studies (1)	/				2.97 (.*84*)	.83
Academic Satisfaction (2)	-.51***	/			3.77 (*1.36*)	.88
Work Centrality (3)	-.10***	.26***	/		3.26 (.*82*)	.78
Self-Efficacy (4)	-.39***	.29***	.17***	/	2.9 (.*51*)	.88
Professional Interest in research (5)	.08**	.16***	.34***	.18***	2.68 (*1.04*)	.92

Note*. For all variables, the skewness and kurtosis were between -1 and +1*

** p <.01

*** p <.001

### Prediction of the intention to pursue an AM career (objective 1)

Ordinal regression models to predict intention to pursue an AM career (objective 1) were built step by step, and for each step, we consider a model with or without controlling for gender. The first model (Model 1) includes the controlled variable of professional interest in research. In the second model (Model 2), background experiences and presence of a mentor (contextual variables) have been added to the previous one. In the third model (Model 3), self-efficacy, outcome expectations and learning experiences (academic satisfaction and perceived stress) have been considered in addition.

Table 4 presents the results of these nested models; note that a predictor is significant when its Odds Ratio (OR) is significantly different from 1, i.e. the confidence interval of its OR does not contain 1. In Model 1, professional interest in research contributed significantly to the prediction of intention to pursue an AM career (without gender R2L = 0.266***, with gender R2L = 0.272***). In Model 2, background experiences and the presence of a mentor contributed significantly to the prediction of intention to pursue an AM career (without gender R2L = 0.033***, with gender R2L = 0.032***). The OR of professional interest in research diminishes but remains the most predictive. In Model 3, work centrality is the only added factor with a significant direct effect on the intention to pursue an AM career.

**Table 4:** Ordinal regression analyses for the block of variables predicting the intention to pursue an AM career

	Without controlling for gender			Controlling for gender			Difference in R2L (model without vs. with gender)
	OR (IC)			OR (IC)			
Model 1							
Professional interest in research	6.4	***	(5.5–7.4)	6.2	***	(5.4–7.3)	
Gender							
Male							
Female				0.6	***	(0.5–0.8)	
R2L	.266***			.272***			.006 ***
Model 2							
Professional interest in research	5.4	***	(4.6–6.3)	5.3	***	(4.5–6.2)	
Background experience							
/							
M1	2.0	***	(1.6–2.6)	2.0	***	(1.6–2.6)	
M2 or PhD	3.6	***	(2.6–5.0)	3.6	***	(2.4–4.9)	
Mentor							
No							
Yes	1.4	**	(1.1–1.7)	1.4	**	(1.1–1.7)	
Gender							
Male							
Female				0.7	***	(0.5–0.8)	
Difference in R2L (model 2 vs. 1)	0.033***			0.032***			.000ns
Model 3							
Professional interest in research	4.9	***	(4.2–5.8)	4.9	***	(4.2–5.7)	
Background experience							
/							
M1	2.1	***	(1.6–2.6)	2.0	***	(1.6–2.6)	
M2 or PhD	3.7	***	(2.7–5.2)	3.7	***	(2.6–5.1)	
Mentor							
No							
Yes	1.3	*	(1.0–1.6)	1.3	*	(1.0–1.6)	
Self-Efficacy	0.9	ns	(0.7–1.2)	0.9	ns	(0.7–1.1)	
Work Centrality	1.5	***	(1.3–1.7)	1.5	***	1.3–1.7)	
Perceived Stress	0.9	ns	(0.8–1.1)	1.0	ns	(0.8–1.1)	
Academic Satisfaction	1.1	ns	(1.0–1.2)	1.1s	ns	(1.0–1.2)	
Gender							
Male							
Female				0.6	***	(0.5–0.8)	
Difference in R2L (model 3 vs. 2)	0.016***			0.016***			.000ns

These results indicate that each addition of predictor blocks contributes to model improvement, whether controlling for gender or not. The weight of professional interest in research diminishes as the steps progress but remains the most substantial contributor by far. This analysis highlights the relevance of better understanding the underlying process of the formation of professional interest in research, which is the most directly determining predictor in the intention to pursue an AM career.

### Process of formation of professional interest in research (objective 2)

**Fig. 2:**
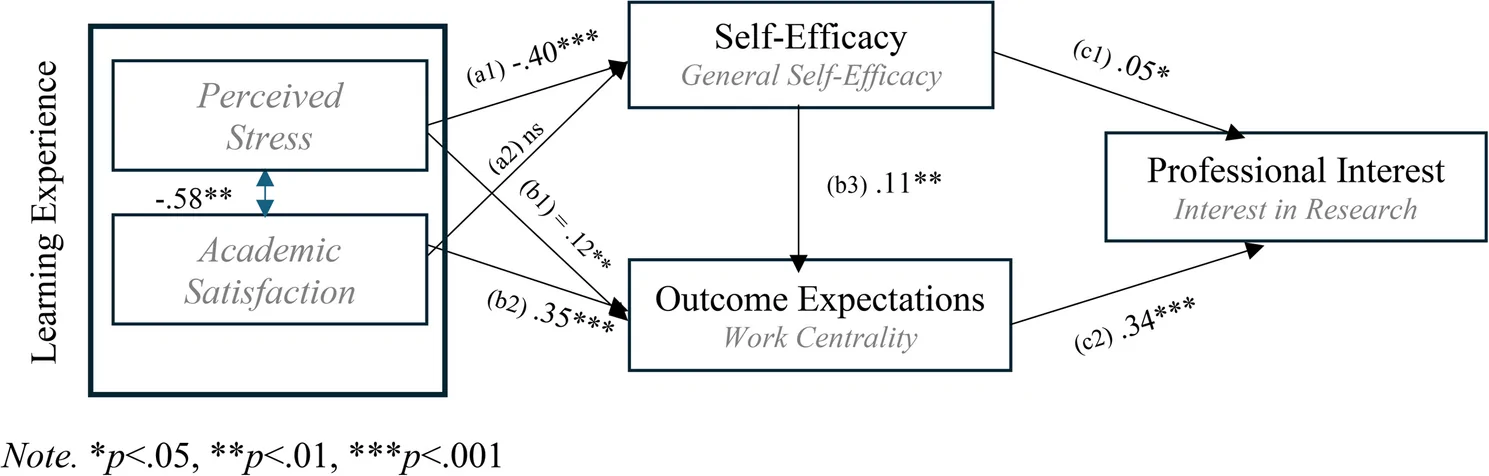
Association between academic satisfaction and work centrality according to gender and research experience

To explore the structural path of professional interest in research, Fig. 2 displays the structural model investigated to predict professional interest as a function of perceived stress, academic satisfaction, self-efficacy and work centrality (objective 2). The structural model is tested controlling for gender and background experiences in all the regressions^2^. The fit index values indicate that the measurement model for perceived stress, academic satisfaction, self-efficacy, work centrality and professional interest in research has good fit to data (CFI = 0.93, TLI = 0.92, RMSEA = 0.04 [0.04-0.05], SRMR = 0.04).

Figure 2 shows that all the paths in the theoretical model are significant. In terms of learning experience, perceived stress has a significant negative effect on self-efficacy (β = − 0.40, *p* < .001) and a positive effect on work centrality (*β* = 0.12, *p* = .008). Academic satisfaction significantly influences work centrality (*β* = 0.35, *p* < .001) but does not have a significant effect on self-efficacy (*p* = .11). Professional interest in research is directly influenced by background experiences, work centrality (*β* = 0.34, *p* < .001), self-efficacy (*β* = 0.05, *p* = .04), and gender (*β* = − 0.08, *p* = .001). Moreover, the influence of self-efficacy on professional interest in research is partially mediated by work centrality (I = 0.04, *p* = .002).

### Moderation of the SCCT interest model by gender and background experience with a multiple-group analysis (Objective 3)

A multiple-group analysis was conducted to examine if the links within an individual’s cognitive process differ according to gender and background experiences (objective 3). Results indicate that gender and background experiences moderate this process (χ² = 58.23, *p* < .01).

Table 5 reveals that, depending on gender, the relationship between work centrality and professional interest in research exhibits no difference in association strength among individuals with no background experiences or those with an M1. However, for those with more advanced degrees such as an M2 or a PhD, the association is stronger for men (*β* = 0.47, *p*<.001) and weaker for women (*β* = 0.33, *p* < .001). Conversely, when examining academic satisfaction and work centrality, a stronger correlation is observed among women with an M2 or a PhD (*β* = 0.42, *p* < .001) compared to men with the same background (*β* = 0.34, *p* < .001).

**Table 5:** Results of the path model depicting SCCT’s prediction of professional interest in research for each crossover between gender and background experience of research

		Women						Men					
		/		M1		M2 or PhD		/		M1		M2 or PhD	
PI:	SE WC	0.04 0.40	ns ***	0.05 0.37	ns ***	0.04 .33	ns ***	0.05 0.39	ns ***	0.04 0.39	ns ***	0.05 0.47	ns ***
SE:	Stress AS	− 0.41 0.06	*** ns	− 0.36 0.06	*** ns	− 0.40 0.07	*** ns	− 0.41 0.06	*** ns	− 0.41 0.07	*** Ns	− 0.41 0.07	*** ns
WC:	SE Stress AS	0.13 0.13 0.38	*** ** ***	0.12 0.11 0.34	*** ** ***	0.13 0.13 .42	*** ** ***	0.12 0.12 0.38	*** ** ***	0.10 0.10 0.31	*** ** ***	0.11 0.11 0.34	*** * ***

*Ns* Non-Significant

*PI *Professional Interest,* SE* Self-Efficacy,* WC* Work Centrality, *AS* Academic Satisfaction

*p<.05; **p<.01; ***p<.001

**Fig. 3:**
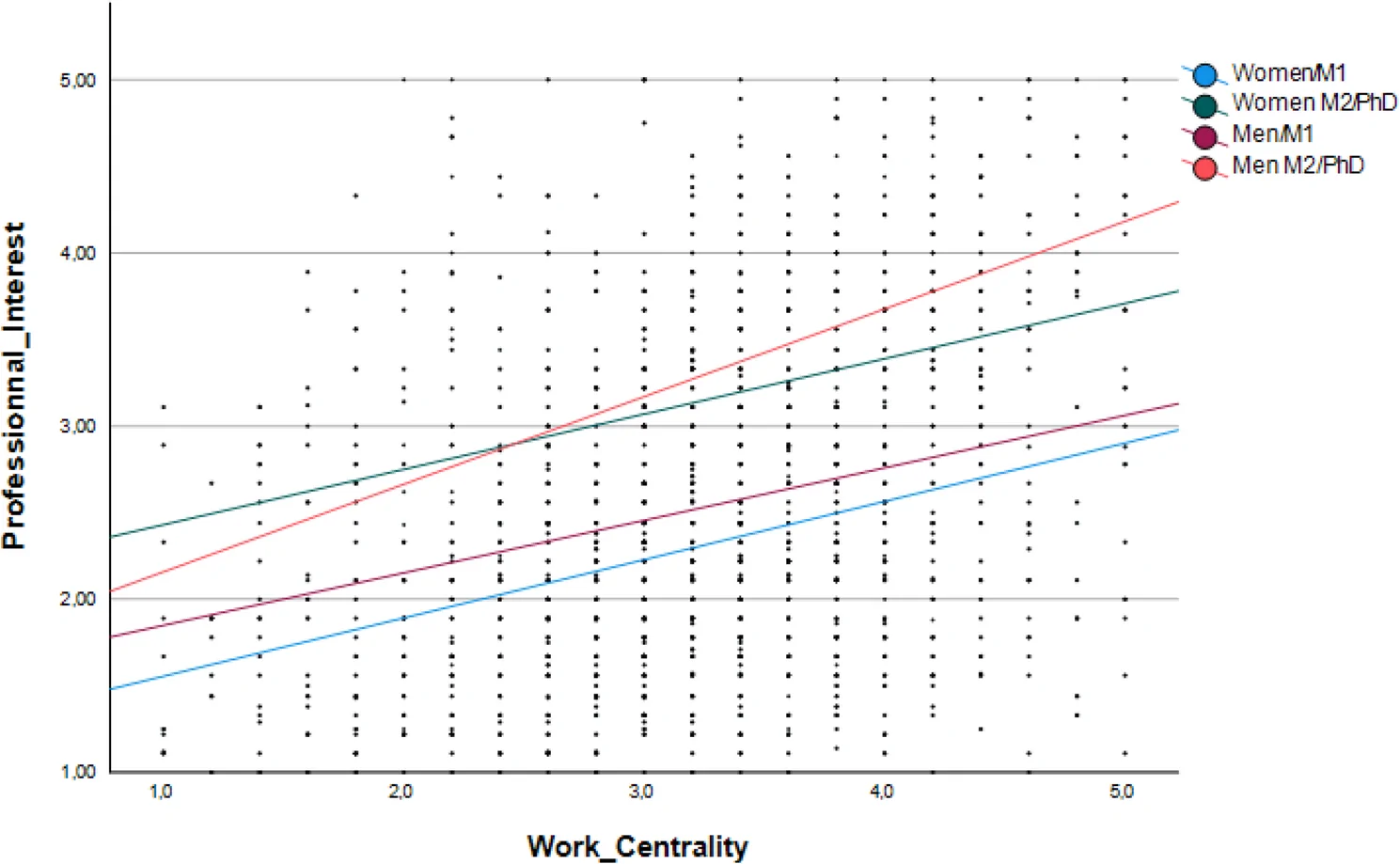
Association between work centrality and professional interest in research according to gender and research experience

**Fig. 4:**
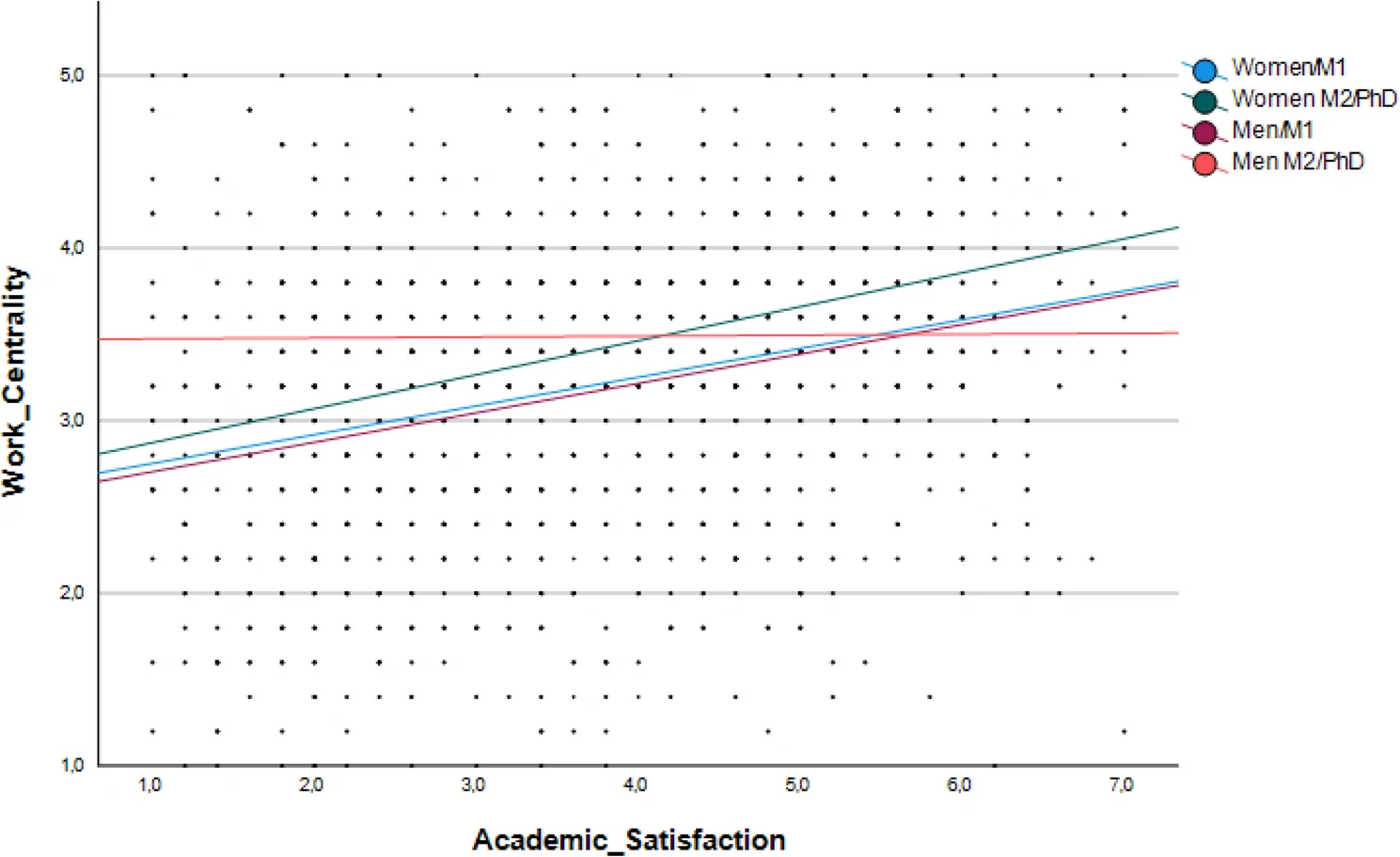
SCCT structural model of professional interest in research, including path coefficient from the full sample test

To facilitate the interpretation of the moderation effects identified in the multigroup analysis, we added graphical representations of the associations between (a) work centrality and professional interest in research (see Fig. 3) and (b) academic satisfaction and work centrality across gender and research experience groups (Fig. 4). These plots are intended as visual illustrations of the group-specific associations reported in Table 5 and should not be interpreted as additional inferential tests. 

## Discussion

This study provides one of the first empirical applications of Social Cognitive Career Theory (SCCT) to academic medicine career intentions among medical residents, particularly within the French context. By integrating cognitive variables and contextual learning experiences, it extends previous research that has typically examined these factors in isolation. In addition, the identification of moderation effects involving gender and research experience offers new insights into the dynamic and potentially stage-dependent nature of academic career development.

Consistent with previous research, professional interest in research emerged as the strongest predictor of intention to pursue a career in academic medicine. This finding is consistent with previous literature highlighting the central role of research interest in academic career choice [4, 5]. While research experiences and mentorship were also significantly associated with this intention, their effects were secondary to the role of interest itself. However, mentorship was assessed using a simplified measure capturing only the presence of a mentor, which does not fully reflect the multidimensional nature of mentorship (e.g., quality, frequency, or types of support). This distinction highlights the importance of differentiating between contextual opportunities (e.g., completing a master’s or PhD) and the cognitive construction of interest in research, as these do not necessarily overlap. Understanding how interest in research develops during medical training therefore appears crucial for addressing academic medicine recruitment challenges.

Outcome expectations, operationalized through work centrality, also played a significant role in predicting AM career intentions. This result highlights the relevance of considering residents’ expectations regarding the personal value and meaning derived from work investment, rather than focusing solely on work–life balance considerations. Academic medicine careers often imply a high level of professional commitment, and research activities may be particularly attractive to individuals who attribute strong personal value to work involvement. In this respect, showcasing inspiring academic role models who find fulfillment in research may contribute to strengthening interest in academic careers.

At the same time, some of the observed relationships warrant cautious interpretation. In particular, the positive association between perceived stress and work centrality may suggest that, in highly demanding training environments such as medical residency, some individuals respond to stress by increasing their investment in work, reflecting a form of adaptive or compensatory engagement. However, this interpretation remains speculative and may reflect context-specific dynamics. Similarly, the absence of a significant relationship between academic satisfaction and self-efficacy may reflect specific features of medical training, where confidence in one’s abilities is shaped more by performance-based experiences than by overall satisfaction with the training environment. More broadly, previous research has highlighted the interplay between self-efficacy, anxiety, and career-related processes, suggesting that these factors may interact in shaping professional trajectories [27].

Gender differences in AM career intentions were largely explained by disparities in learning experiences, research opportunities, and professional interest in research. While gender initially contributed to the prediction of AM career intention when professional interest was considered alone, its effect diminished once contextual variables such as research experiences and mentorship were included. This pattern suggests that structural and environmental factors may play a more important role than individual preferences in explaining women’s underrepresentation in academic medicine. This interpretation is consistent with previous literature highlighting the role of structural barriers and organizational factors in explaining gender disparities in academic medicine [7, 11]. Recent evidence further shows that, although women physician-scientists achieve similar success at early career stages, they are significantly less likely to progress to independent investigator roles, reflecting structural and institutional barriers rather than higher attrition from academia [28]. Notably, no gender differences were observed in outcome expectations as measured by work centrality, indicating that women and men hold similarly strong expectations regarding the value of work investment.

Moderation analyses further suggest that gender differences may emerge at more advanced stages of research involvement. Among residents with an M2 or a PhD, the association between work centrality and professional interest in research was stronger for men than for women. This finding suggests that, at advanced levels of research training, the extent to which professional commitment and the value attributed to work translate into interest in research may differ according to gender. Such patterns are consistent with evidence showing that gender disparities become more pronounced at advanced stages of academic careers, particularly in access to independent research funding [29]. This finding points to potential gender-specific turning points in the career decision-making process and highlights the need to better understand how advanced research experiences are perceived and integrated into career trajectories, particularly for women.

These differences may reflect gender-specific ways of interpreting and integrating advanced research experiences into career decision-making processes. For example, while such experiences may reinforce alignment with academic career trajectories for some individuals, they may also highlight structural constraints or perceived barriers for others, particularly among women. This interpretation remains exploratory and calls for further research to better understand the mechanisms underlying these moderation effects.

From a medical education perspective, these findings emphasize the importance of promoting supportive learning environments during residency. Reducing perceived stress, enhancing academic satisfaction, and providing early and structured exposure to research activities may be especially beneficial for fostering interest in academic careers. In the French context, this could involve facilitating access to research training during residency (e.g., protected research time, support for Master’s or PhD programs) and strengthening the visibility and accessibility of academic career pathways. Integrating research experiences into medical curricula and ensuring access to mentorship could represent key institutional strategies to strengthen engagement in research, particularly among women. In addition, developing more structured mentorship initiatives within hospital-university settings may help support residents’ career development and reduce existing disparities. These recommendations are consistent with recent work emphasizing the need for coordinated institutional strategies to promote gender equity in academic medicine [30].

Overall, these findings are in line with previous research while providing a more integrated perspective through the SCCT framework.

## Limitations and conclusion

Several limitations should be acknowledged. First, the cross-sectional design of the study precludes causal interpretations of the observed relationships. Longitudinal research would be valuable to capture the dynamic evolution of learning experiences, cognitive variables, and career intentions throughout residency.

Second, some core SCCT constructs were operationalized using measures that may not fully capture their domain-specific or multidimensional nature. In particular, self-efficacy was assessed using a general, cross-domain measure rather than a domain-specific measure of research self-efficacy, which may have attenuated the observed associations. Outcome expectations were operationalized through work centrality, reflecting a general orientation toward work rather than expectations about the specific consequences of engaging in research activities, as defined within SCCT. This conceptual mismatch may have led to an underestimation of the role of outcome expectations in shaping professional interest in research. In addition, mentorship was assessed using a single dichotomous item, which does not capture its multidimensional nature (e.g., relationship quality, types of support). Taken together, these measurement limitations may have reduced the precision with which SCCT processes were modeled. Future studies should incorporate more domain-specific and multidimensional measures to better reflect the theoretical constructs.

An additional limitation relates to the recruitment strategy and the absence of a known response rate, which may have introduced self-selection bias and potentially limit the representativeness and generalizability of the findings. This issue is further compounded by the characteristics of the sample, particularly the high proportion of participants without children. While this may reflect the relatively young age of medical residents in France and the tendency to delay parenthood during demanding training periods, we did not collect detailed data on family structure or caregiving responsibilities to support this interpretation. As such, the extent to which this distribution accurately reflects the broader population of French medical residents remains uncertain.

In conclusion, this study extends existing literature by providing a novel application of SCCT to academic medicine career intentions among medical residents in France. By highlighting the central role of professional interest in research and integrating both cognitive and contextual determinants, it offers a more comprehensive understanding of academic career development. The identification of gender-specific moderation patterns further contributes to the literature by suggesting that the mechanisms underlying career intentions may vary depending on the level of research engagement. These findings provide actionable insights for medical education institutions seeking to promote academic medicine careers and address gender disparities. While focused on academic medicine, this integrative and context-sensitive approach may also be relevant to academic career pathways more broadly.

## Supplementary Information


Supplementary Material 1.


## Data Availability

In line with principles of transparency, openness and reproducibility, de-identified synthetic data and statistical analysis code supporting the findings of this study are publicly available on the Open Science Framework (OSF) at [https://osf.io/cmeb8/?view_only=994c4caf23c744858f00a1260469395a](https:/osf.io/cmeb8/?view_only=994c4caf23c744858f00a1260469395a).

## Footnotes

1 Île-de-France refers to the Paris metropolitan area, the most densely populated region in France.

2 Without controlling for gender and background experiences, the relationship between self-efficacy and professional interest in research as well as the relationship between work centrality and professional interest in research were stronger.
