# Supplementary material for: Academic medicine career intentions among medical residents: a social cognitive career theory approach
Source: BMC Med Educ. 2026 Jun 19;26:1405. doi: 10.1186/s12909-026-09740-4 (PMC13531863; doi:10.1186/s12909-026-09740-4)
Supplement: Supplementary file 1 [file 12909_2026_9740_MOESM1_ESM.docx]

# Questionnaire – Medical Career Choices

# *(English version)*

## Information

Thank you for agreeing to participate in this survey on career choices in medicine.

The aim of this questionnaire is to gather your most sincere responses to a series of questions regarding your experience of health-related studies, as well as your aspirations and choices concerning your professional orientation.

There are no right or wrong answers: only your honest responses are of interest to us. All your responses will be treated confidentially.

In this study, we are interested in your thoughts, emotions, and behaviors across a wide range of domains related to your future career choices.

## Informed Consent to Participate

In order to make an informed decision, I have received and understood the following information:

1. I have been informed that my participation is entirely voluntary and that I am free to accept or refuse to take part in the study.
2. I may withdraw from the study at any time without having to provide justification and without incurring any consequences.
3. The data collected concerning me will be strictly confidential and anonymous and will be processed electronically in accordance with applicable data protection regulations.
4. My consent does not release the study sponsor from any of its responsibilities, and I retain all rights guaranteed by law.
5. I may access the overall results of the study once it has been completed, upon request by email to a member of the research team (projet-pulwar@univ-nantes.fr).
6. I may request additional information at any time from a member of the research team (projet-pulwar@univ-nantes.fr).

I agree to participate in the study on medical career choices and authorize the members of the research team to use all data concerning me that are collected as part of this study.
[YES / NO]

## Career Project

What is your medical specialty?
*[Drop-down menu with a list of specialties]*

What is your affiliated university?
*[Drop-down menu with a list of universities]*

In which year did you take the national ranking examination at the end of the second cycle of medical studies (ECN)?

What was your ranking at the ECN?

Which year of residency are you currently enrolled in?
*[Drop-down menu]*

Have you completed a Master’s degree (Master 1)?
*(Yes / No / Ongoing)*

Have you completed a Master’s degree (Master 2)?
*(Yes / No / Ongoing)*

Have you completed a PhD?
*(Yes / No / Ongoing)*

Have you already participated in research activities (studies, article writing, research protocols)?
*(Never / Rarely / Sometimes / Often)*

To what extent do you wish to pursue an academic career as an academic hospital physician (assistant or full professor)?
*(From not interested at all to very interested)*

Your future career plans:
*(Multiple answers possible: exclusively clinical activity; combined clinical, teaching and research activity; possible career change; other)*

## Demographics

Gender:
*Male / Female / Non-binary*

Age (in years):

Do you have children?
*(No / 1 child / 2 or more children)*

Mother’s occupational category:

Father’s occupational category:

If you are in a relationship, partner’s occupational category:

## Satisfaction with Studies

The following statements relate to your satisfaction with your studies. For each statement, please indicate your level of agreement on a scale from 1 (*strongly disagree*) to 7 (*strongly agree*).

- In general, my studies are close to my ideal.
- My study conditions are excellent.
- I am satisfied with my student life.
- So far, I have obtained the important things I wanted from my studies.
- If I could start my current training program again, I would change almost nothing.

## Stress Assessment

The following questions aim to assess the level of stress you experienced during your studies. Please respond to each statement using a scale ranging from 1 (*almost never*) to 5 (*always*).

- During your studies, how often did you feel that things were going your way?
- During your studies, how often did you feel confident in your ability to handle your personal problems?
- During your studies, how often did you feel that you were unable to control the important things in your life?
- During your studies, how often did you feel that difficulties were piling up so high that you could not overcome them?

## Sense of Competence (Self-efficacy)

You are now invited to complete a questionnaire assessing how you perceive yourself in your daily life (behaviors, reactions). For each statement, please indicate your response on a scale from 1 (*not at all true*) to 4 (*completely true*).

- I can always manage to solve difficult problems if I try hard enough.
- If someone opposes me, I can find means and ways to get what I want.
- It is easy for me to stick to my aims and accomplish my goals.
- I am confident that I could deal efficiently with unexpected events.
- Thanks to my resourcefulness, I know how to handle unforeseen situations.
- I can solve most problems if I invest the necessary effort.
- I can remain calm when facing difficulties because I can rely on my coping abilities.
- When I am confronted with a problem, I can usually find several solutions.
- If I am in a bind, I can usually think of something to do.
- No matter what comes my way, I’m usually able to handle it.

## Research and You

The following questions aim to assess your attitudes toward research. Please indicate your response on a scale from 1 (*strongly disagree*) to 5 (*strongly agree*).

- Research is indispensable in my professional life.
- I enjoy studying current research results.
- I cannot think of life without research.
- I do not find research problems interesting.
- I like doing research.
- Research boosts my career confidence.
- I do not think research will produce any results.
- I like topical research.
- Research improves general knowledge.

**Importance Attached to Work (Work Centrality)**

The following statements relate to what you would like for your future professional life. For each statement, please indicate your level of agreement on a scale from 1 (*strongly disagree*) to 5 (*strongly agree*).

- I want my work to provide me with satisfaction and a sense of fulfillment, as I intend to devote a large part of my time to it.
- I feel that my sense of self-worth will come from my work and the strength of my commitment
- In the future, even if I did not need money, I cannot imagine myself not working.
- I desire for my work to shape my identity and radiate into all areas of my life
- I hope that work will remain central in my life.

## Perception of Mentoring

In your professional environment, do you have a mentor (a knowledgeable and experienced guide or advisor, someone you particularly respect, whom you can take as a role model, and/or who provides you with expertise and experience)?
[YES / NO]

Mentor’s status:
Academic hospital physician (assistant professor / full professor) or other
(equivalent to MCU-PH / PU-PH in the French system)

## Conclusion

Do you think that an academic hospital career is accessible to everyone?
(Yes / No)

Why?
(Open-ended response)
